# Listeriosis outbreak likely due to contaminated liver pâté consumed in a tavern, Austria, December 2018

**DOI:** 10.2807/1560-7917.ES.2019.24.39.1900274

**Published:** 2019-09-26

**Authors:** Adriana Cabal, Franz Allerberger, Steliana Huhulescu, Christian Kornschober, Burkhard Springer, Claudia Schlagenhaufen, Marianne Wassermann-Neuhold, Harald Fötschl, Peter Pless, Robert Krause, Anna Lennkh, Andrea Murer, Werner Ruppitsch, Ariane Pietzka

**Affiliations:** 1 Institute for Medical Microbiology and Hygiene, Austrian Agency for Health and Food Safety, Vienna/Graz, Austria; 2 European Public Health Microbiology Training Programme (EUPHEM), European Centre for Disease Prevention and Control (ECDC), Stockholm, Sweden; 3 Department – Health and Nursing Management, Styrian Provincial Government, Graz, Austria; 4 Section of Infectious Diseases and Tropical Medicine, Department of Internal Medicine, Medical University of Graz, Graz, Austria

**Keywords:** *Listeria monocytogenes*, outbreak, Austria, liver pâté, whole genome sequencing

## Abstract

In late December 2018, an outbreak of listeriosis occurred after a group of 32 individuals celebrated in a tavern in Styria, Austria; traditional Austrian food (e.g. meat, meat products and cheese) was served. After the celebration, 11 individuals developed gastrointestinal symptoms, including one case with severe sepsis. Cases had consumed mixed platters with several meat products and pâtés originating from a local production facility (company X). Human, food and environmental samples taken from the tavern and company X were tested for *L. monocytogenes.* Whole genome sequence-based typing detected a novel *L. monocytogenes* strain of serotype IVb, sequence type 4 and CT7652 in 15 samples; 12 human, two food and one environmental sample from company X with an allelic difference of 0 to 1. Active case finding identified two further cases who had not visited the tavern but tested positive for the outbreak strain. In total, 13 cases (seven females and six males; age range: 4–84 years) were identified. Liver pâté produced by company X was identified as the likely source of the outbreak. Control measures were implemented and since the end of December 2018, no more cases were detected.

## Background

*Listeria monocytogenes* is a Gram-positive bacterium that is typically transmitted to humans through the consumption of contaminated food products. Clinical symptoms of listeriosis vary depending on the immune status of the host, with those immunocompromised at higher risk of presenting severe symptoms [1]. In immunocompetent individuals, infection can be asymptomatic but it more often results in febrile gastroenteritis [2]. Febrile gastroenteritis usually resolves within 2–3 days after the onset of symptoms, while invasive forms of the disease can lead to meningoencephalitis, abortion, sepsis or even death [3,4]; other manifestations such as endophthalmitis have also been associated to infections with *L. monocytogenes* [1,5]. Foods that have been implicated with listeriosis outbreaks are ready-to-eat (RTE) products such as sliced meat, pâté and soft cheese varieties [6]. Since *L. monocytogenes* can persist in the environment for long periods due to its ability to form biofilms and its resistance to disinfectants, this pathogen can be difficult to eradicate from food-processing facilities [7].

In Austria, notification of invasive listeriosis cases is mandatory. The Austrian National Reference Laboratory for Listeria (NRL; Graz Austria) is responsible for performing whole genome sequence (WGS)-based typing of human and non-human (e.g. food, environmental) isolates. In recent years, WGS-based surveillance of *L. monocytogenes* has been successfully used in combination with analysis of epidemiological data in outbreak investigations [8-10].

### Outbreak detection

On 21 December 2018, the local health authority of Styria (Directorate of Public Health, Graz, Austria) and the NRL confirmed the occurrence of an outbreak of febrile gastroenteritis, including one case of culture-confirmed *L. monocytogenes* bacteraemia, among 32 persons having attended a tavern in the province on 15 December 2018 as part of a celebration. Previously, a pregnant physician who had visited the tavern and was aware that *L. monocytogenes* had been isolated from a blood culture of a tavern guest had informed the local health authority of Styria about a possible listeriosis outbreak. According to her, more than half of the guests started showing symptoms of febrile gastroenteritis and vomiting within 2 days after the tavern visit.

On 29 January 2019, the Austrian Ministry of Health (Vienna, Austria) mandated the Austrian Agency of Health and Food Safety (AGES; Graz, Austria) to investigate the outbreak. The aim of the investigation was to identify the causative agent and the likely source of infection, in order to detect and prevent further cases.

## Methods

### Outbreak case definition

An outbreak case was defined as an individual who presented with febrile gastroenteritis at least 24 hours after visiting the tavern in Styria, Austria on 15 December 2018 and tested positive for *L. monocytogenes* by either blood or stool culture.

### Specimen collection and trace-back investigations

Of 32 individuals attending the celebration on 15 December 2018, 19 symptomatic individuals provided stool specimens. Of five individuals working in this tavern on this day, three asymptomatic individuals provided stool samples. The local food authority gathered information on involved food suppliers, restricting it to one local meat producer (company X) and three grocery shops (A, B and C).

Between 3 January and 25 January 2019, a total of 73 food and environmental samples were analysed for the presence of *L. monocytogenes:* (i) 19 environmental (surface) samples and three food samples produced by company X were collected from the tavern, (ii) three samples were collected from food produced at other facilities (shops A to C) (iii) one sample was collected from food produced by the tavern itself, and (iv) 47 food and environmental samples were collected from company X at their production facility. Products from company X were only offered at six locations, including the affected tavern and sold directly from the factory. 

### Microbiological investigations and WGS-based typing

The detection of *L. monocytogenes* in human samples was conducted as described elsewhere [11] and colonies were confirmed by Api-*Listeria* (BioMérieux, Marcy l'Etoile, France) or Maldi Biotyper (Bruker Daltonics, Hamburg, Germany). Food and environmental isolates received at the NRL were cultivated on RAPID'L.mono agar plates (Biorad, Munich, Germany) for species verification and subsequently subcultured overnight on Columbia Broth (BD Difco, Heidelberg, Germany) for extraction of high quality genomic DNA using the HMW MagAttract kit (Qiagen, Hilden, Germany) according to the instructions of the manufacturer for Gram-positive bacteria.

WGS was performed as described previously [12]. For sequencing, an Illumina MiSeq platform (Illumina Inc., San Diego, California, United States) was used. Library preparation was carried out using Nextera XT according to the instructions of the manufacturer (Illumina Inc.). For assembly into draft genomes, raw reads were de novo assembled using SPAdes version 3.11.1 [13]. Classical multilocus sequence typing (MLST) data were extracted from WGS sequence data [14]. A minimum spanning tree (MST) was generated in SeqSphere + for visualisation of strain relatedness. Assessment of the core-genome multilocus sequence typing (cgMLST) was done as described by Ruppitsch et al. [8]. In parallel, a single nucleotide polymorphism (SNP) analysis was performed for comparison purposes with GenomeGraphR [15]. In addition, we created two task templates that were implemented with Seqsphere + for detection of the pathogenicity islands LIPI-3 and LIPI-4 using the reference genomes from strain F2365 and strain CLIP 80459, respectively (NC_002973.6, NC_012488.1). Other virulence genes (VGs) were detected among the sequenced genomes by using VirulenceFinder [9].

### Active case finding

We conducted active case finding to detect additional human cases (retrospectively and prospectively). A new case definition was created, which included any person testing positive to the outbreak strain by WGS since three months before the tavern gathering. We performed an intensive search and strain comparison using our local *L. monocytogenes* database, which currently contains nearly 8,000 sequenced isolates.

### Ethical statement

Ethical approval to conduct the study was not needed. In Austria, investigation of foodborne outbreaks is a legal obligation.

## Results

### Microbiological findings

Of 19 stool specimens collected from individuals with febrile gastroenteritis, 10 samples (specimen ID: H03–H10, H12, H14) were positive for *L. monocytogenes.* Of three stool specimens from asymptomatic staff members of the tavern, only one stool sample from a female in her early 60s (H11) tested positive for *L. monocytogenes*. In addition, one *L. monocytogenes* isolate came from a blood culture obtained from a male in his mid-20s who was hospitalised for febrile gastroenteritis 24 hours after the tavern visit (H02).

Of 73 non-human samples, two food samples and one environmental sample tested positive for *L. monocytogenes*. The two food samples (specimen ID: F01: smoked meat (‘Geselchtes’) and F02: liver pâté (‘Leberstreichwurst’) were collected at the tavern and produced by company X; both tested positive for *L. monocytogenes* in 25 g of product in concentrations of < 10 colony-forming unit (CFU)/g. A gully water sample (F03), obtained in January 2019 by the Styrian health authorities at the production facility of company X also tested positive for *L. monocytogenes*.

**Figure 1:**
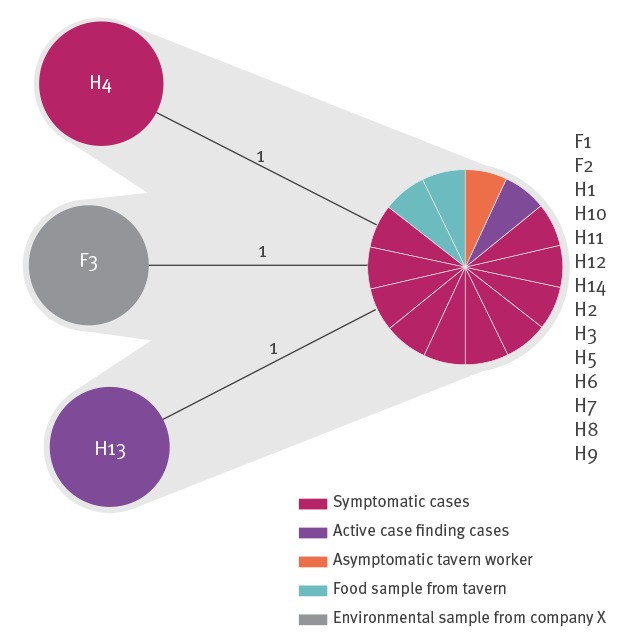
Minimum spanning tree representing the genetic relatedness among the 17 sequenced isolates based on their core genome, Styria, Austria, 2018

In addition to the three non-human isolates (F01–F03), 12 human *L. monocytogenes* isolates (from specimens: H2–H12, H14) were available for sequencing. cgMLST performed as described by Ruppitsch et al. [8] revealed that all isolates belonged to the same genetic type: they were identified as genoserogroup IVb, clonal complex (CC)4, multilocus sequence type (ST)4 and cg complex type (CT)7652 [8] and displayed 0 to 1 allelic difference (Figure 1). In total, 1,701 loci composing the cgMLST scheme were detected. The SNP analysis revealed a maximum of four SNPs difference between the isolates and we did not find related strains in the database. All isolates yielded the same 72 virulence genes including LIPI-3 and LIPI-4 pathogenicity islands.

Isolates show 0 to 1 alleles of difference. Accession numbers can be consulted in Supplementary Table S1.

### International alert and active case finding

Since this outbreak strain had not been detected previously in Austria, an urgent inquiry was launched through the Epidemic Intelligence Information System (EPIS) at the European Centre for Disease Prevention and Control (EPIS-UI-539) on 28 January 2019. Denmark, Finland, France, the Netherlands, Luxembourg, Portugal, Switzerland and the United Kingdom answered the inquiry via the EPIS platform; no cases were linked to this outbreak strain. The closest matches were found at the European Nucleotide Archive repositories showed at least 32 allelic differences when using the cgMLST scheme from Moura et al. [10].

Through active case finding, we identified two more cases of invasive listeriosis. These cases lived in the same two geographical districts (‘Bezirk’) as the tavern and company X. The first isolate was taken from a blood culture from a case in their early 80s (H1) who had not visited the tavern but developed symptoms in November 2018. The case, who later died from the infection, reported having repeatedly consumed liver pâté from company X purchased at a local market. The second isolate originated from an eye chamber aspirate from a case in their mid-50s with endophthalmitis (H13), with onset of clinical symptoms on 23 December 2018; the case had not visited the tavern. The two isolates differed by a maximum of one allele from the other clinical isolates and the food and environmental isolates.

In total, 13 individuals (seven females and six males; age range: 4–84 years) living within area radius of 24 km in Styria, were confirmed as outbreak cases.

**Figure 2:**
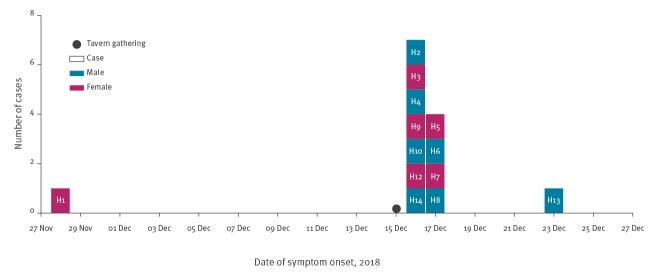
Cases of listeriosis, by date of symptom onset, Styria, Austria, 2018 (n = 13)

**Figure 3:**
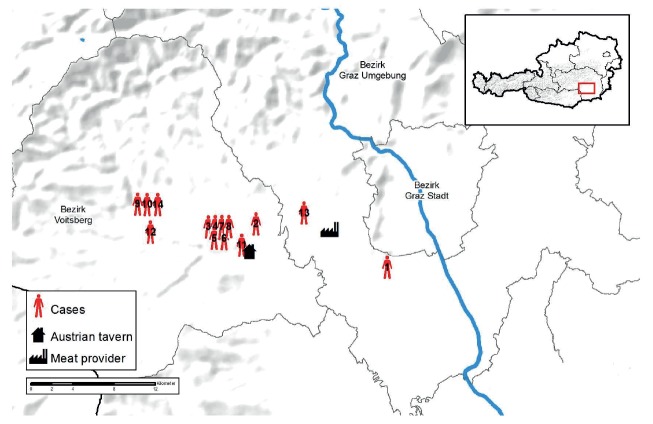
Map showing the geographical location of confirmed cases, the tavern and company X, Styria, Austria, 2018

A timeline displaying all cases belonging to the outbreak is shown in Figure 2. A geographical representation of the residence for the confirmed *L. monocytogenes* cases, the tavern and the meat producer company X is shown in Figure 3.

An asymptomatic tavern worker (H11) is not depicted in the timeline. Eleven cases were tavern cases and two additional cases matching the second case definition were identified by active case finding.

### Outbreak control measures

After the outbreak was confirmed on 21 December 2018, intensive cleaning of the tavern and at company X was performed by professional sanitation companies, under supervision of the local health authority. Only heat-treated products were allowed to be sold until confirmed sanitation (i.e. *L. monocytogenes* was no longer detected after repeated sampling). No more cases were detected by the end of December and the outbreak was declared over.

## Discussion

The use of epidemiological data and WGS-based typing allowed us to confirm a local outbreak due to an *L. monocytogenes* IVb-CC4-ST4-CT7652 strain, not previously detected in Austria nor anywhere else. Trace-back investigations showed that meat products originating from company X were the most likely source of the outbreak. The 13 confirmed cases lived within a radius of 24km, further suggesting that the outbreak was caused by a locally sourced ingredient. No more cases were detected after company X implemented control measures, supporting the hypothesis that meat products from company X were the likely source of the outbreak.

In contrast to classical restaurants, a tavern offers a limited selection of traditional food (especially local meat products and cheese) in a buffet-like manner but has a fully functioning kitchen including a fridge and a freezer. In Austria, gatherings of large groups at traditional taverns are common around festivities such as Christmas. It is possible that the time between food preparation and service might have been long increasing the possibility for *L. monocytogenes* to multiply. Moreover, traditional Austrian meat products e.g. liver pâté and jellied pork do not require heating prior to consumption and have been previously identified as potential risk food for listeriosis due to their growth potential for *L. monocytogenes* [16,17]. In 2009, an outbreak of febrile gastroenteritis was reported that was also associated with a traditional Austrian tavern, this time locally produced jellied pork was identified as the vehicle of infection [11]. Liver pâté (‘Leberstreichwurst’) is a peculiar type of traditional Austrian meat product, made from cured, cooked, comminuted meat with fat as a binder. Its average pH value is 6.19 ± 0.15, with a corresponding water activity value of 0.963 ± 0.003, which provides *L. monocytogenes* with an optimal substrate for its growth [17]. Other types of pork pâté such as ‘paté de campagne’ were found to be contaminated with *L. monocytogenes* [18]. Although enumeration of *L. monocytogenes* in the pâté seemed low (< 10 CFU/gram), no Tween 80 was added to its initial suspension during the enrichment procedure, and that might explain the relatively low number of bacteria found per gram.

The only case without a proven connection to liver pâté of company X was the case with endophthalmitis; only 43 cases of endophthalmitis attributed to an *L. monocytogenes* infection have been reported in the literature so far [5,19-21]. While we were unable to elucidate any connection of this patient to company X, the case confirmed regular consumption of liver pâté so it is possible that the meat product may have been produced at company X.

*L. monocytogenes* serotype IVb strains are commonly associated with outbreaks [22]. Moreover, serotype IVb CC4 is known to be widely distributed in food and it has been described as one of the IVb hypervirulent CCs [23,24]. All 14 human isolates (from the 13 confirmed cases plus the stool isolate from the asymptomatic case), the two food isolates and the environmental isolate carried LIPI-3 and LIPI-4 pathogenicity islands, which have been associated with high invasiveness and severe symptomatology [25]. In contrast, another article reported the hypervirulent CC4 was associated with dairy products, while hypovirulent clones, such CC9 or CC121 were associated with meat products [26].

The main limitation of our study was associated with the closure of the tavern over the Christmas period. Due to the closure, the AGES outbreak investigation did not start until 45 days after the tavern visit. Nevertheless, control measures had been previously put in place preventing new cases to occur.

### Conclusion

The isolate-based surveillance of *L. monocytogenes* using WGS-based typing and analyses of epidemiological data allowed us to confirm a local outbreak due to a *L. monocytogenes* IVb-CC4-ST4-CT7652 strain not previously detected elsewhere. Epidemiological and trace-back investigations showed the liver pâté produced at company X was the most likely source of infection. The applied control measures were effective in stopping the outbreak. Additional investigations are needed to estimate the risk of infection with *L. monocytogenes* when attending celebrations at taverns serving non-heated products that have been stored at room temperature for extended periods of time.

## Depositing of the sequences

This whole-genome shotgun project has been deposited in DDBJ/EMBL/GenBank under the project number PRJNA528854 and corresponds to the study number SRP189297. Raw sequence data for each strain were deposited under SRA accession numbers SRR8776907, SRR8776908, SRR8776904, SRR8776910, SRR8776917, SRR8776915, SRR8776918, SRR8776920, SRR8776906, SRR8776905, SRR8776911, SRR8776912, SRR8776913, SRR8776914, SRR8776916, SRR8776919, SRR8776909.

## Supplementary Data

144000 Supplementary Material S1
