# Supplementary material for: Listeriosis outbreak likely due to contaminated liver pâté consumed in a tavern, Austria, December 2018
Source: Euro Surveill. 2019 Sep 26;24(39):1900274. doi: 10.2807/1560-7917.ES.2019.24.39.1900274 (PMC6774228; doi:10.2807/1560-7917.ES.2019.24.39.1900274)
Supplement: Supplementary Material S1 [file 19-00274_ROSEL_SupplementaryMaterialS1.pdf]

Supplementary material

Disclaimer: This supplementary material is hosted by Eurosurveillance as supporting information alongside the article “Listeriosis outbreak likely due to contaminated liver pâté consumed in a tavern, Austria, December 2018” on behalf of the authors who remain responsible for the accuracy and appropriateness of the content. The same standards for ethics, copyright, attributions and permissions as for the article apply. Supplements are not edited by Eurosurveillance and the journal is not responsible for the maintenance of any links or email addresses provided therein.

Table S1. SRA accession numbers of the corresponding sequenced isolates (project PRJNA528854, study SRP189297).

Isolate ID	SRR Accession
H-01	SRR8776907
H-02	SRR8776908
H-03	SRR8776904
H-04	SRR8776910
H-05	SRR8776917
H-06	SRR8776915
H-07	SRR8776918
H-08	SRR8776920
H-09	SRR8776906
H-10	SRR8776905
H-11	SRR8776911
H-12	SRR8776912
H-13	SRR8776913
H-14	SRR8776914
F-01	SRR8776916
F-02	SRR8776919
F-03	SRR8776909
